# Native synthetic microbial communities enhance zha‐chili by boosting the fermentation capacity of indigenous microorganisms

**DOI:** 10.1002/imo2.70009

**Published:** 2025-03-16

**Authors:** Hongye Shen, Chuanyu Du, Shu Jiang, Weiwei Dong, Jinshan Li, Yongmei Hu, Nan Peng, Shumiao Zhao

**Affiliations:** ^1^ National Key Laboratory of Agricultural Microbiology and College of Life Science and Technology Huazhong Agricultural University Wuhan China; ^2^ College of Life Sciences Hubei Normal University Huangshi China

**Keywords:** flavoring substance, microbial community succession, synthetic community, traditional fermented foods, zha‐chili

## Abstract

Fermented foods are a crucial part of the global diet, accounting for one‐third of global food intake. Traditional fermented foods often rely on natural fermentation, leading to safety risks. The construction of synthetic microbial communities (SynComs) tailored for fermented foods is a key strategy to solve these issues. Here, we designed and constructed SynComs consisting of two bacterial and three fungal species, utilizing the study model of zha‐chili. Using various high‐throughput sequencing technologies, the dynamic alternations of microorganisms during the fermentation process were investigated, and the impact of SynComs on the fermentation process was evaluated. SynComs reduced fermentation time by approximately 15 d, increased flavor yields (8% for ethyl lactate and ethyl acetate), and greatly improved the quality of the zha‐chili. Meanwhile, SynComs altered the succession of the fungal community so that Pichia became the dominant microorganism throughout the fermentation process, and the pattern of fungal community succession was brought closer to the null model. Metagenomic annotation results showed notable changes in functional genes, especially in glycoside hydrolases family. SynComs enhanced the positive correlations between indigenous microorganisms and flavor compounds while increasing other community microorganisms' contribution to flavor production. These findings provide a new approach to improve the quality of zha‐chili and other traditional fermented foods through natural fermentations. We proposed that SynComs enhanced fermented foods by boosting the fermentation capacity of indigenous microorganisms.

## Highlights

- A native synthetic microbial community suitable for traditional fermented foods—zha‐chili, was constructed to increase the flavor content and shorten the fermentation time of zha‐chili products, and it was verified that the synthetic microbial community functioned better than a single bacterial inoculation.
- The synthetic microbial community significantly changed the fungal community succession in zha‐chili, but had little impact on the bacterial community succession.
- The synthetic microbial community may improve the quality of zha‐chili by enhancing the contribution of other native microorganisms in the community to aroma substances.

## Graphical abstract

Traditional fermented foods in synthetic microbial communities (SynComs). We simulated the production of zha‐chili, a traditional fermented food, in the laboratory, intending to address the following questions: How are SynComs constructed, and what is the effect of inoculation with SynComs on the quality of zha‐chilli? How does inoculation with SynComs affect microbial community succession?

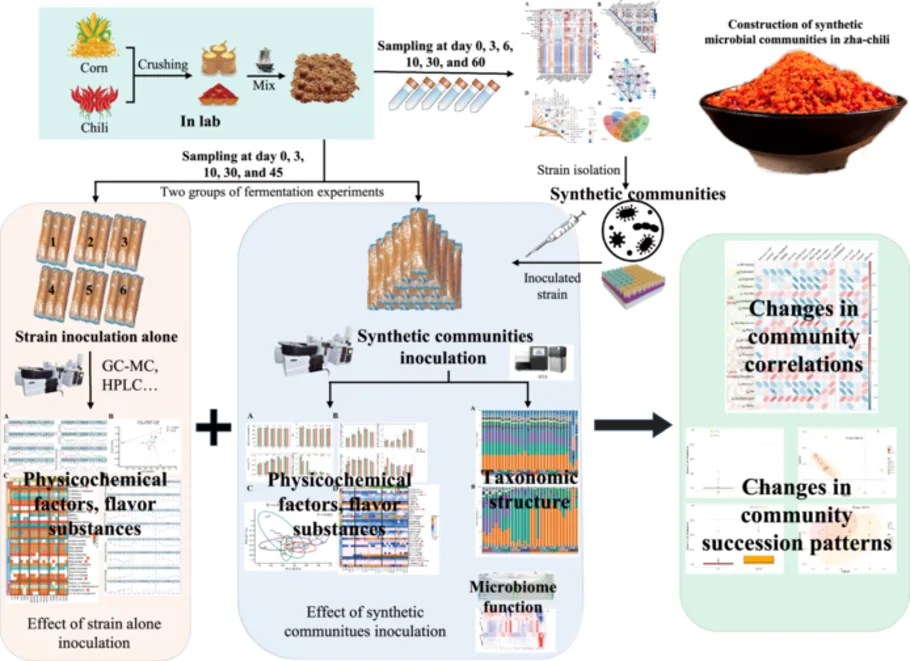

## 1 INTRODUCTION

The world is currently facing escalating challenges in nutrition and food security [1, 2, 3, 4]. Fermented foods are crucial to the global food supply, enriching diets, improving gut microecology, and promoting human health. Fermentation is an ancient method of food preservation, with the earliest bread‐based products dating back to 14,600 BC [5]. The activity of the microbial community during fermentation determines the safety, nutritional value, and yield of fermented foods. Many fermented foods involve the introduction of specific microorganisms during preparation. For example, Chinese baijiu adds jiuqu, many Japanese fermented foods use Koji starter, and lactic acid bacteria are added to kefir [6, 7, 8]. However, many traditionally fermented foods do not introduce specific fermentation flora, relying solely on environmental microorganisms and those introduced by the producers, which is commonly observed in less developed regions of Southeast Asia and Africa [9, 10]. With the advancement of industrialized production, naturally fermented traditional foods encounter issues such as food safety and inconsistent product quality [11, 12, 13]. Inoculating with beneficial microorganisms contributing to fermentation represents a promising solution to these problems.

Synthetic microbial communities (SynComs), developed at the intersection of synthetic biology and microbial strain engineering, bring new opportunities for fermented foods production [14, 15, 16]. SynComs not only broadens the range of microbial foods that can be engineered but also enables the production of safe and healthy fermented foods or feeds. Despite their application in several fields, the screening and validation methods for the effectiveness of SynComs in fermented foods are not widely recognized. Most current studies do not thoroughly investigate how SynComs affect the fermentation micro‐ecosystem and the extent of their effects [17, 18, 19, 20]. Additionally, experimental designs to validate the effects of individual strains within SynComs are insufficient. Research focused on the construction, application, and validation of SynComs, as well as the exploration of their roles and mechanisms in fermented food production, remains limited. By comparing the effects of SynComs inoculation with single‐strain inoculation, we can elucidate how SynComs interact and their impact on the microecological environment. This will provide a crucial theoretical basis and practical guidance for optimizing fermented food production processes and enhancing product quality and safety. Understanding the dynamic changes in microbial community structure and function during fermentation is fundamental to control the fermentation system [21]. Microorganisms in fermented foods originate from diverse sources and species, primarily falling into three major groups: bacteria, filamentous fungi, and yeasts. These microorganisms play distinct roles in fermentation, forming a complex microbial community. *Lactobacillus*, *Acetobacter*, *Bacillus*, and *Clostridium* spp. are predominant among the bacteria. These bacteria contribute to the unique taste and nutritional value of fermented foods by breaking down starches and producing organic acids. *Lactobacillus*, in particular, not only breaks down starches but also inhibits the growth of pathogenic bacteria, which is essential for ensuring the safety of fermented foods [22, 23]. Yeasts, such as *Saccharomyces cerevisiae*, produce esters, alcohols, and other flavoring substances from organic acids [24, 25, 26]. These substances contribute unique flavors and nutritional value to fermented foods, enhancing their appeal. Filamentous fungi, such as *Trichoderma*, *Rhodophyta*, and *Trichoderma*, primarily produce a variety of extracellular enzymes [27]. These enzymes, including proteases, amylases, pectinases, cellulases, and polyphenolases, significantly impact the quality and taste of fermented foods. However, due to the diversity of microorganisms in fermented foods, it is not feasible to inoculate and fortify all microbial types. Therefore, it is crucial to construct the SynComs that significantly enhance fermentation quality. This will not only improve the quality and safety of fermented products but also facilitate the industrialization and automation of fermented food production [28].

Metagenomics‐based approaches have been successfully used to characterize the composition and function of microorganisms in various ecological environments [29, 30]. However, there are fewer metagenomics applications in fermented foods; current studies focus on changes in the relative abundance of metagenomics and environmental factors during fermentation, with a lack of studies on changes in functional microbial genes [31, 32]. Metagenomics provides more accurate species identification and can reveal the potential functional genes of microbial communities, thus rendering it an effective method to analyze the dynamic patterns of change in the structure and function of biological communities. This method facilitates a deeper understanding of the successional changes in fermentation microbial communities.

Here, we simulated zha‐chili production in the laboratory (Figure S1A) and collected samples from the fermentation process for multiple assays and analyses, aiming to address the following questions: (1) Can analytical and computational methods be used to obtain SynComs and isolate the target strains from multiple batches of zha‐chili through amplicon sequencing and assays for various flavoring substances (Figure S1B)? (2) How do the strains act individually (Figure S1C), and what is the impact of SynComs inoculation on the taxonomy and function of the microbiome throughout the fermentation process (Figure S1D)? Is the group inoculated with SynComs superior to the control group and the group in which each strain acts alone? (3) How does the inoculation of SynComs affect microbial community succession during zha‐chili fermentation, and what is the effect of SynComs on other microorganisms in the community (Figure S1E)? Furthermore, this study aims to propose that SynComs improve the entire microbial system by altering the functions performed by other microorganisms. This study comprehensively investigated the construction and functional evaluation of SynComs in fermented foods, improved the quality of zha‐chili products, and provided new research insights for the control and enhancement of other fermented foods or simpler microbiological environments.

## 2 RESULTS

### 2.1 SynComs screening in zha‐chili

**FIGURE 1:**
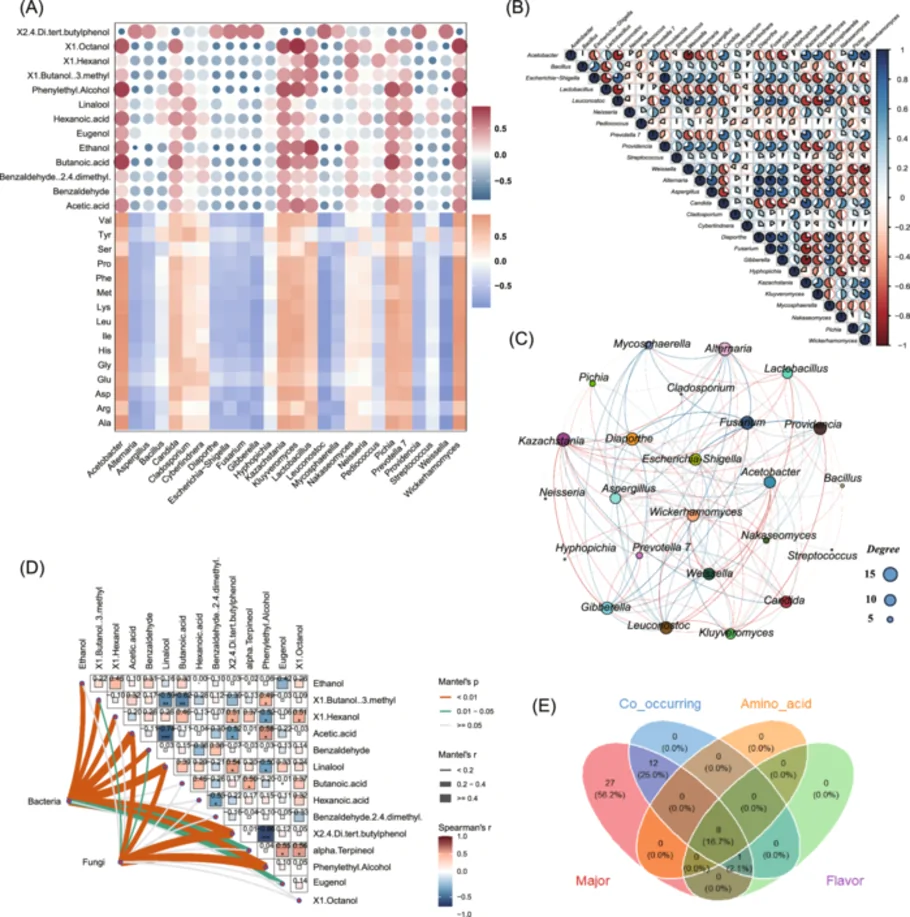
Screening for functional microbial genera. (A) Correlation heat map of major microbial genera, volatile flavor substances, and free amino acids. (B) Correlation heat map between major microorganisms. (C) Network analysis between major organisms. (D) Mantel correlation analysis of bacterial and fungal communities and major flavor substances. (E) Statistically significant synthetic microbial communities (SynComs) obtained by combining the abilities of various aspects of the genus.

To select SynComs suitable for zha‐chili fermentation microbiome, we extensively studied the dynamics of the microbiome, flavor compound changes, and physicochemical factors during zha‐chili fermentation (0, 3, 10, 30, and 45 days). Correlation analysis revealed two key microbes significantly influencing zha‐chili quality: the Flavor Group, which produces volatile flavor compounds, and the Amino Acid Group, responsible for free amino acid production (Figure 1A). In addition, we employed direct correlation analysis and microbial network analysis to identify strong nodal microbes in the zha‐chili fermentation system, known as coincident populations (Figure 1B,C), Flavor group, Amino acid group, Co‐occurring group, and Major group containing strains in Table S1. These microorganisms play a vital role in the fermentation process, as their stability and activity greatly influence fermentation quality. Mantel correlation analysis revealed the importance of synergy between bacteria and fungi in chili fermentation (Figure 1D). They exhibited strong associations with a wide range of flavor substances and physicochemical factors, suggesting that the SynComs should contain both bacteria and fungi. Based on these analyses, we defined the Flavor group, Amino acid groups, Co‐occurring groups, and Major groups (average relative abundance > 0.1%). We further cross‐referenced these groups to identify the SynCom1, which showed statistical significance (Figure 1E).

To ensure food safety, we screened indigenous microorganisms in situ from zha‐chili to form SynComs. To fulfill this goal, three rounds of zha‐chili products were screened to isolate many native organisms. It is worth noting that amino acid producing yeasts with high relative abundance (e.g., *Kazachstania* (45%) and *Kluyveromyces* (0.8%)) in the yeast group were not obtained as culturable strains, but *Saccharomyces* (0.09%*)* was isolated in three batches and was of orders of magnitude between 10^4^ and 10^6^. *Saccharomyces* had been considered to be an important flavor‐producing yeast in fermented foods [24], then we identified *Saccharomyces* as an alternative zha‐chili aroma‐producing yeast. Combining the statistically significant SynCom1 and strain isolation results, we identified *Lactobacillus brevis* (LAB), *Weissella confusa* (Wess), *Pichia kudriavzevii* (Pichi), *Candida glabrata* (Can), and *Saccharomyces cerevisiae* (Sac) as SynComs in this study. This approach integrates the results of biological analyses and the isolation of indigenous strains to obtain SynComs that contribute to the optimization and quality of the zha‐chili fermentation process.

### 2.2 Fermentation effect of strains alone in SynComs

Before the inoculation of fermentation by SynComs, this study investigated the role of individual strain inoculation to test whether inoculation of a single strain was sufficient to improve the quality of zha‐chili. The activity of microbial communities frequently elicits alterations in various physicochemical factors that reflect the metabolic activity and function of the microbial community [33, 34, 35].

**FIGURE 2:**
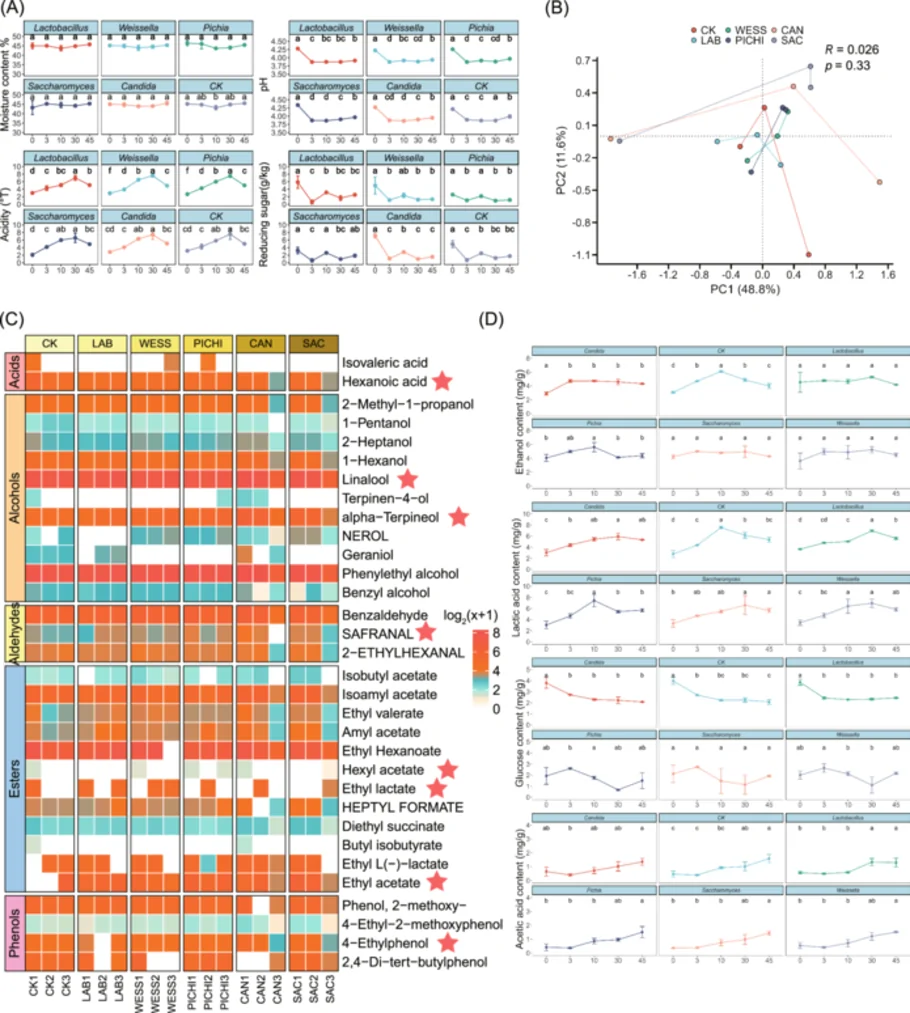
Effect of individual inoculation of strains in a SynComs. (A) Changes in major physicochemical factors during fermentation of zha‐chili in separate inoculation experiments. (B) Results of principal component analysis (PCA) of volatile flavor substances. (C) Heat map of the relative content of major volatile flavor substances in each group, with the stars indicating the common flavor substances in food. (D) Changes in the content of major organic acids, ethanol, and glucose in each group.

By assaying the alterations in water content, acidity, and pH during the fermentation process, the samples from the six groups showed a predominantly uniform pattern of changes (Figure 2A). However, regarding changes in reducing sugar content, the two groups of samples inoculated with Pichi‐1 and Sac‐1 had significantly lower reducing sugar content at the beginning of fermentation (Day 0) than the other four groups. Although the changing trend was consistent, with reducing sugar content gradually decreasing to its lowest point from 0 to 3 days, then slightly rebounding from 3 to 10 days, and continuing to fluctuate and decrease after that, these two groups of samples had a lower initial reducing sugar content. As for organic acids, the important flavoring substances in fermented foods, lactic acid, acetic acid, and ethanol showed similar changing modes across all six groups. It is noteworthy that the contents of these substances did not differ obviously between the six groups at the end of fermentation. Notably, the three groups of samples inoculated with Wess‐1, Pichi‐1, and Sac‐1 exhibited lower reducing sugar or glucose contents at the beginning of fermentation (Day 0), indicating that these strains utilize sugar more efficiently at the initial stage of fermentation.

Assisted with the gas chromatography‐mass spectrometry (GC‐MS) analysis, we identified over 140 volatile metabolites in zha‐chili products from six different experimental groups at the end of fermentation. Subsequent analysis of variance employing principal component analysis (PCA) revealed that intra‐group differences were smaller in the WESS, LAB, and PICHI groups, indicating that the fermented products from these three groups exhibited heightened similarity concerning volatile flavor substance content and product quality was relatively stable (Figure 2B). From these 140 volatile flavor substances, we screened out more than 30 with a pleasant aroma classified as edible flavors. These flavor substances were classified into five categories based on their chemical composition: acids, alcohols, aldehydes, esters, and phenols. The volatile flavor substances of particular interest, as indicated in Figure 2C, were given particular attention. Of particular note within the flavor esters, ethyl lactate, and ethyl acetate have been identified as prevalent components in fermented foods. Ethyl lactate was higher in the LAB and WESS groups, while ethyl acetate was found to be lower in the CK group. Hexanoic acid was slightly lower in the SAC and CAN groups. Additionally, linalool and nerol, which are naturally occurring flavor compounds with a floral aroma, were found in similar amounts in all six groups, while nerol was abundant in the WESS and PICHI groups compared to the other groups. Alpha‐terpineol, a constituent of numerous plant essential oils, was found in similar amounts in all six groups. Notably, safranal, the main active substance in saffron providing a distinct odor and aroma, was found in the highest levels in the PICHI group and lower levels in the CK and CAN groups.

Overall, single‐organism inoculation increased the yield of some volatile flavor substances, although the trends of the main physicochemical factors did not change with individual strain inoculation. However, a singular strain cannot effectively enhance the yield of the main flavor substances of zha‐chili.

### 2.3 Effect of inoculation with SynComs on the quality and microbiological composition of zha‐chili

**FIGURE 3:**
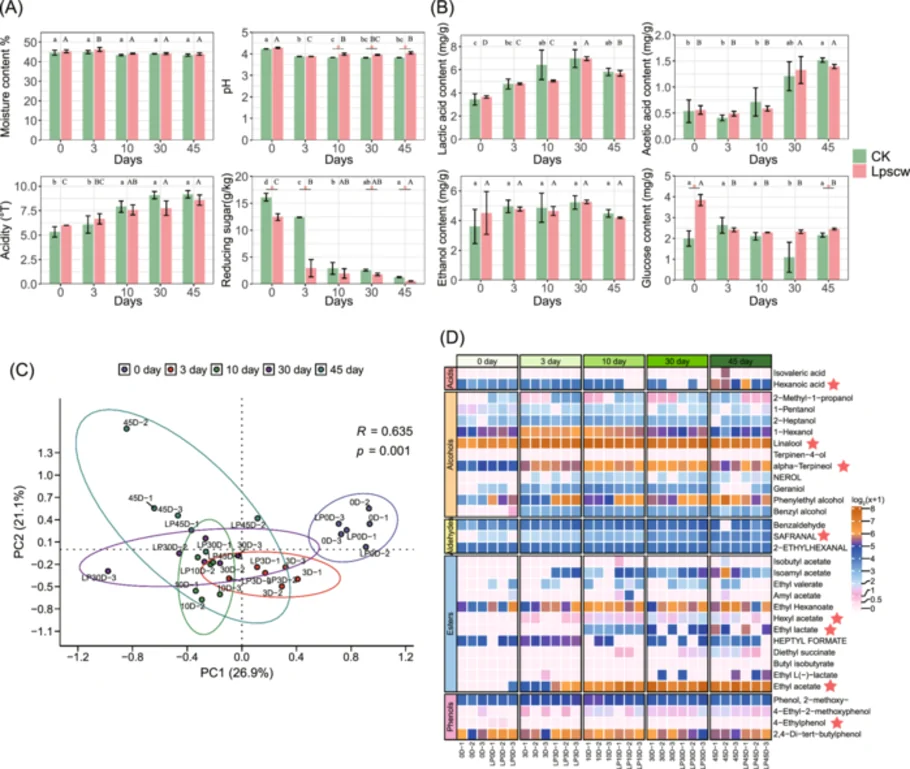
The effectiveness of SynComs inoculation. (A) Changes in major physicochemical factors during fermentation of zha‐chili in separate inoculation experiments. (B) Changes in the content of major organic acids, ethanol, and glucose in each group. Significant differences in factors over time are indicated by lowercase in the CK group and uppercase in the Lpscw group. (C) PCA analysis of volatile flavor substances. (D) Heatmap of the relative content of major volatile flavor substances in each group, with the stars indicating the common flavor substances in food. PCA, principal component analysis.

In this study, we compared the changes between the control group (CK group) and the group inoculated with SynComs (Lpscw group) during fermentation. Changes in major physicochemical factors, flavor substances, microbial community composition, and functional genes were monitored and quantified at five time points: Days 0, 3, 10, 30, and 45 (Figure 3A–D). The CK and Lpscw groups demonstrated analogous trends in water content and acidity; however, the Lpscw group exhibited a more rapid decrease in pH and reduced sugar content during the early fermentation period (Days 0–3) (Figure 3A). Lactic acid content continued to rise in both groups from 0 to 30 days, peaking on Day 30; acetic acid content accumulated from 10 to 30 days, while ethanol content remained stable throughout the entire fermentation process. Notably, the glucose content of the Lpscw group was significantly higher than that of the CK group at the beginning of fermentation, and the glucose content of the CK group fluctuated more throughout the fermentation process (Figure 3B).

**FIGURE 4:**
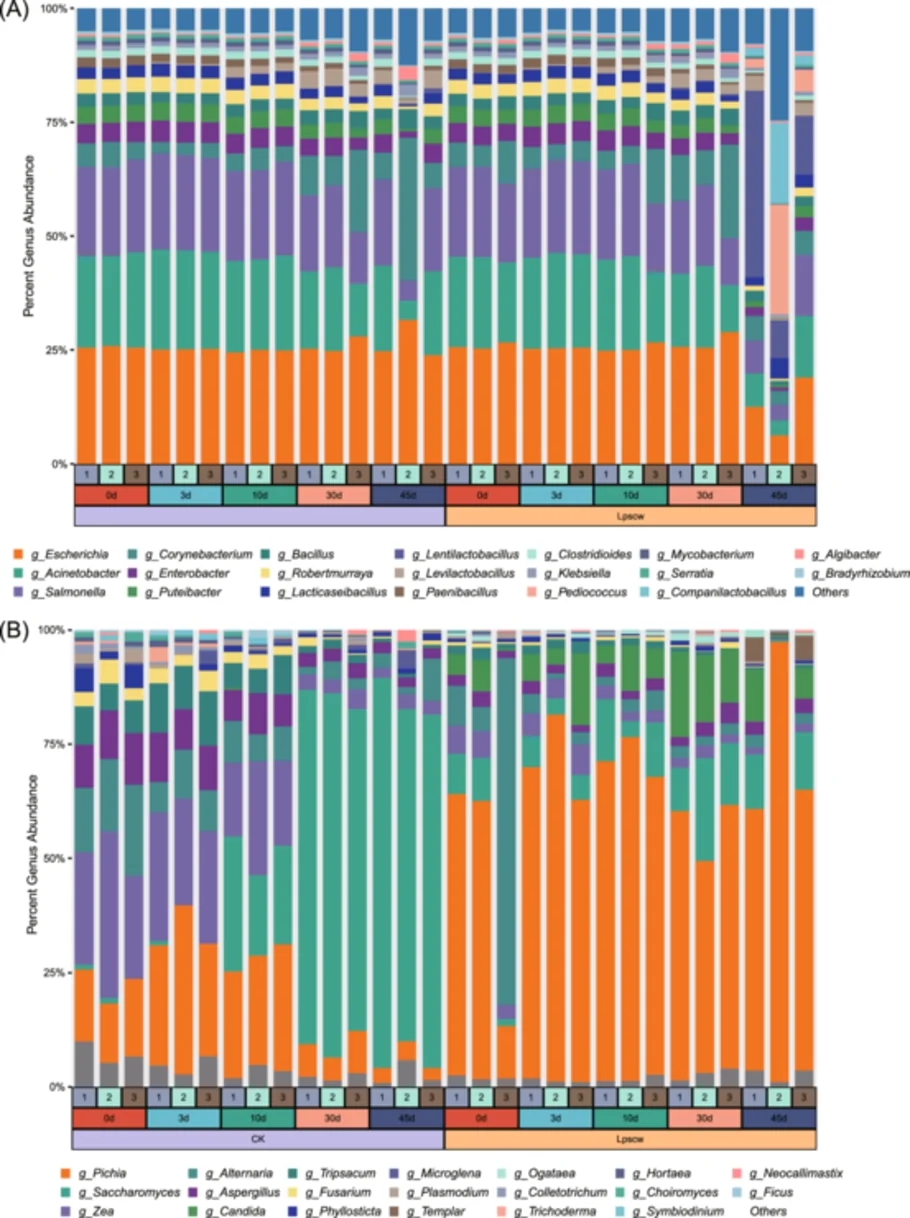
Composition and dynamic of the microbial community at the genus level (fungal top 20, bacterial top 20), the numbers represent repeated samples in each region. (A) The dynamic of bacterial fungal community. (B) The dynamic of fungal community at four sites.

The GC‐MS assay showed that the differences between the CK and Lpscw groups were greater than the intra‐group differences (*R* = 0.6353, *p* = 0.001), and samples from different fermentation times could be effectively distinguished (Figure 3C). During the early stage of fermentation (0–10 days), the flavor substances were similar in the two groups; however, as fermentation progressed to 30–45 days, the differences in flavor substances between the Lpscw and CK groups gradually increased. The content of most volatile compounds increased over fermentation time, but the content of a few compounds decreased (Figure 4D). In particular, the contents of specific flavor substances (e.g., linalool, nerol, safranal, geraniol, ethyl lactate, and ethyl acetate) were generally higher in the Lpscw group than in the CK group at the later stages of fermentation (30–45 days). Additionally, the Lpscw group produced less hexanoic acid (with odor) than the CK group at the late fermentation stage, suggesting that the Lpscw group was superior in producing more flavoring substances and fewer odorous substances. In conclusion, the Lpscw group outperformed the CK group in all aspects of the fermentation process.

This study utilized metagenomics sequencing technology to comprehensively trace the succession process of microbial communities in the Lpscw and CK groups during the fermentation of zha‐chili at the bacterial genus level. The microbial composition and successional processes of the two groups showed similar trends (Figure 4A), with *g_Escherichia*, *g_Acinetobacter*, *g_Salmonella*, and *g_Corynebacterium* representing the main dominant bacterial genera. Due to the additional inoculation of *Lacticaseibacillus* in the Lpscw group, the mean abundance of *g_Lacticaseibacillus* (2.5%) was observed to be marginally higher in Lpscw group than in the CK group (2.33%). However, despite the addition of *Weissella*, its abundance in the bacterial community remained outside the top 50. At the fungal genus level (Figure 4B), the main dominant fungal genera were similar in the Lpscw and CK groups, including *g_Pichia*, *g_Saccharomyces*, *g_Zeag*, *g_Alternaria*, *g_Aspergillus*, and *g_Candida*. However, the changes in community succession of fungal genera showed significant differences between the two groups. In the CK group, *g_Pichia* and *g_Zeag* dominated during the pre‐fermentation period (0–3 days) but were gradually replaced by *g_Saccharomyces* as fermentation progressed. In contrast, in the Lpscw group, *g_Pichia* maintained its dominance throughout the entire fermentation period. Furthermore, *g_Candida* became one of the dominant genera with a total average abundance of 8.5% in the Lpscw group, compared to only 0.05% in the CK group.

Linear discriminant analysis effect size (LEfSe) analyses further revealed species (biomarkers) with significant differences between the two groups (Figure S2). Among bacteria, the relative abundance of biomarkers was generally higher in the Lpscw group and was predominantly of the genus *Pediococcus*, possibly due to additional inoculation of *Lactobacillus*. Among the fungal biomarkers, the most prevalent were from the genus *Pichia* and the order Saccharomycetes, with *Pichia_kudriavevii* being one of the strains added to the Lpscw group, leading to its naturally higher abundance. However, it is noteworthy that all other fungal biomarkers, except *Pichia_kudriavevii*, also had generally higher abundance in the Lpscw group, suggesting that SynComs altered the fungal community succession during the fermentation of zha‐chili. Overall, inoculation with SynComs significantly affected fungal community succession during the fermentation of zha‐chili but a lesser effect on the composition of bacterial community.

### 2.4 Effect of inoculation with SynComs on functional genes of zha‐chili microbial community

Functional gene annotation was carried out using the Kyoto Encyclopedia of Genes and Genomes (KEGG) and carbohydrate‐active enZYmes (CAZy) databases in this study. The results of the KEGG database annotation demonstrated that metabolism was the predominant annotation result in both groups, and the other five major pathways accounted for less than 5% of the total (Figure S3A,B). Among the metabolic pathways, the metabolism of carbohydrates, amino acids, and lipids occupied the highest proportion. In particular, the carbohydrate metabolism was the most dominant function of the microbial community in zha‐chili. The CAZy database annotation results revealed that the glycoside transferases (GHs) family was the most dominant, accounting for more than 60% of the CAZymes, followed by glycoside hydrolases (GHs) at about 12%, while carbohydrate esterases (CEs) and carbohydrate‐binding modules (CBMs) accounted for a smaller share. The top 10 major enzymes associated with carbohydrate degradation in the Lpscw and CK groups included GT1, GH28, GH38, PL4, CE6, GT2, GH19, GH17, CBM50, and GH36 (Figure S3B). GT1, the most abundant enzyme in the fermentation microbial community, is responsible for the biosynthesis of glycosidic bonds from phosphate‐activated sugar donors. As fermentation progresses, the abundance of GT1 gradually decreases, suggesting that the need for glycosyltransferases decreases in the later stages of fermentation. GH28, GH38, and GH19 belong to the glycoside hydrolyzing group of enzymes, including glycosidases and transglycosidases, which are responsible for either hydrolysis or transglycosylation of glycosidic bonds. In the CK group, the abundance of GH28, GH38, and GH19 continued to increase during fermentation; however, in the Lpscw group, their abundance increased (0–30 d) and then decreased (30–45 d), which may be attributed to the fact that sugar metabolism in the Lpscw group was completed within 30 days.

Based on LEfSe analysis, we compared the functional gene annotation results of the KEGG and CAZy databases between the Lpscw and CK groups (Figure S4). In KEGG, the CK group exhibited greater activity in functions such as translation, ribosomes, propionate metabolism, and betaine biosynthesis, while the Lpscw group displayed higher activity in starch, sucrose, glycine, serine, and threonine metabolism. Both groups demonstrated high abundance in several functions, highlighting the complexity of the fermentation process. In the CAZy database, the biomarker specific to the Lpscw group primarily involved the GT and GH families. These variations may affect carbohydrate metabolism and product diversity. This suggests that the inoculation of SynComs obviously altered CAZyme functional genes during zha‐chili fermentation, potentially influencing carbohydrate metabolism and the diversity of metabolites.

### 2.5 Effect of inoculation with SynComs on zha‐chili microbial community construction patterns and community microbial functions

**FIGURE 5:**
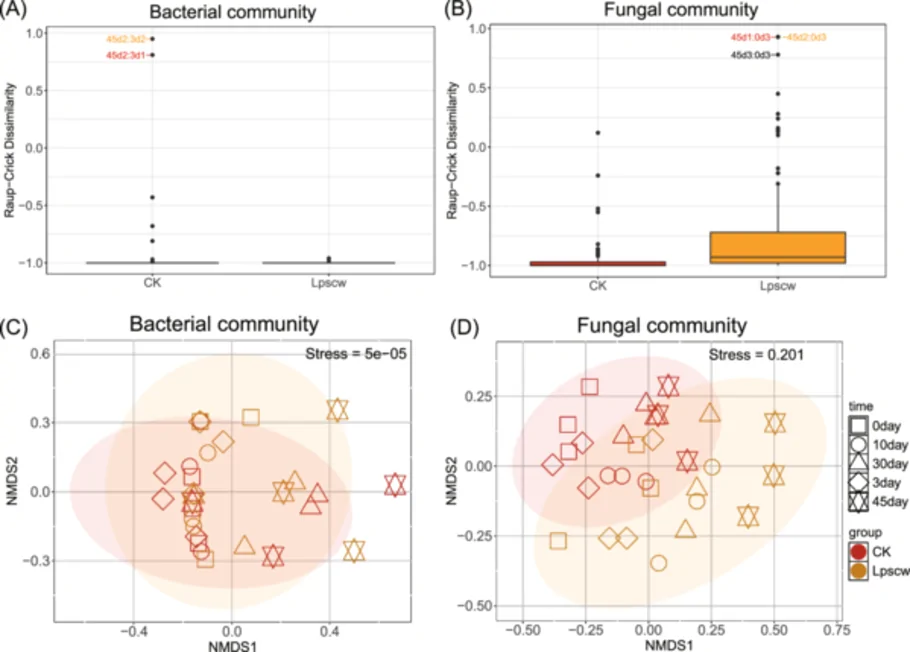
Calculated Raup–Crick index analyses between Lpscw and CK group communities. (A) Raup–Crick index distribution of the bacterial community between groups. (B) Raup–Crick index distribution of the fungal community between groups. (C) Nonmetric multidimensional scaling (NMDS) ordering of communities based on the Raup–Crick dissimilarity index for the bacterial community between groups. (D) NMDS ordering of communities based on the Raup–Crick dissimilarity index for the fungal community between groups.

Modern ecological theories generally agree that microbial community assembly is shaped by both deterministic and stochastic factors. Deterministic processes follow the ecological niche theory, where selection and competition driven by environmental pressures play significant roles. In contrast, stochastic processes follow the neutral theory and are influenced by dispersal and random births and deaths. Using the Raup‐Crick index (RC index) for evaluation, we observed that the RC index of the Lpscw bacterial community in zha‐chili approached −1, indicating a high similarity among bacterial communities primarily driven by deterministic processes, particularly ecological niche selection. While the RC index of the bacterial community in the CK group also approached −1, notable differences were observed (Figure 5A). Regarding fungal community succession, RC indices in the CK group similarly approached −1, suggesting that fungal communities were less variable and more similar than expected randomly, predominantly under deterministic influences. However, in the Lpscw group, fungal RC indices exhibited a varied pattern, with some nearing 0, indicating similarity or dissimilarity aligned with neutral model expectations. Notably, samples from Day 45 in the Lpscw group diverged from other samples obviously (Figure 5B). Nonmetric multidimensional scaling (NMDS) analyses revealed that early and mid‐stage zha‐chili bacterial communities clustered together, indicative of deterministic processes, whereas late‐stage communities exhibited dispersion, suggesting stochastic influences (Figure 5C,D). Overall, the fungal community exhibited strong environmental selection and deterministic filters, as indicated by the NMDS plots, with stress values below 0.2, confirming the robustness of the analyses.

**FIGURE 6:**
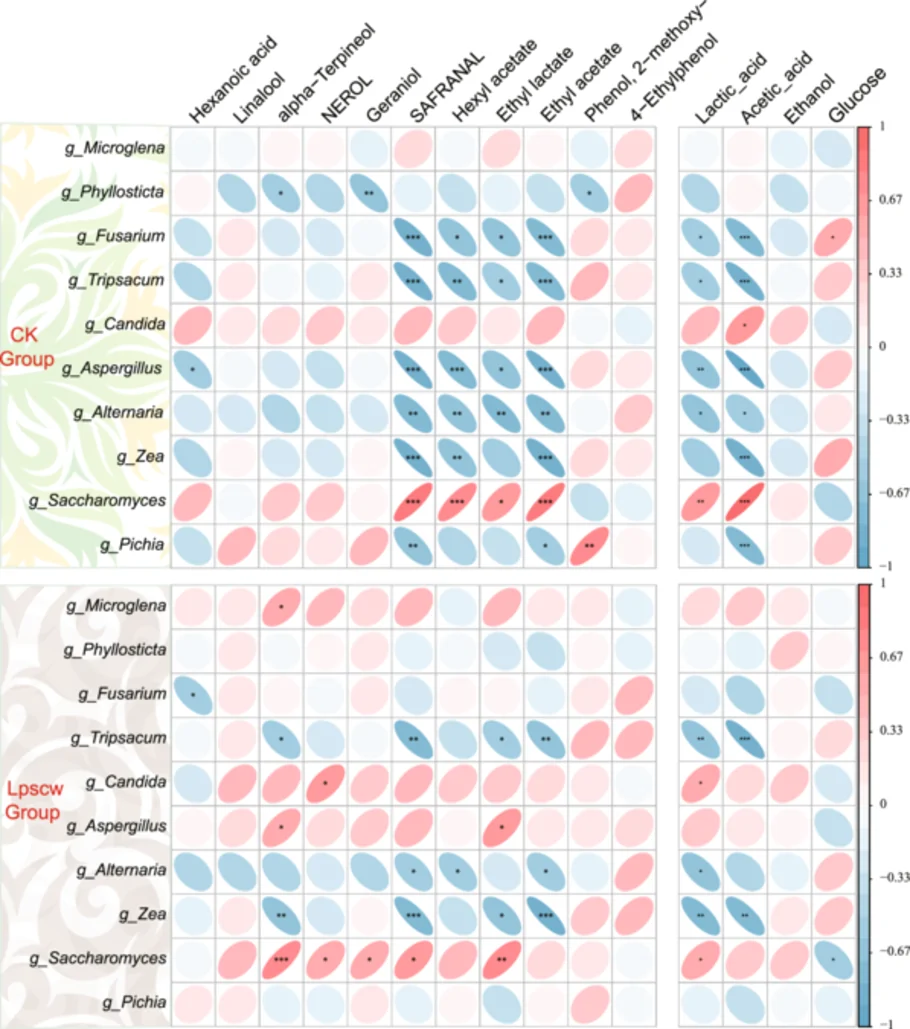
Correlation results between Lpscw and CK groups with major volatile flavor substances and major organic acids. The top 10 fungi in terms of relative abundance and the main flavor substances were selected for detailed analysis. (* means *p* < 0.05, ** means *p* < 0.01, *** means *p* < 0.001.)

The quality of zha‐chili is strongly influenced by its production of volatile flavor substances. In the CK group, *Pichia*, *Saccharomyces*, and *Candida* exhibited significant positive correlations with major flavor substances, consistent with preliminary sequencing findings (Figure 6). Specifically, *Pichia* exhibited significant positive correlations (*p* < 0.01) with higher alcohols such as Linalool, Nerol, alpha‐Terpineol, Geraniol, and 4‐Ethyl‐2‐methoxyphenol, a phenol analog. *Saccharomyces* showed significant positive correlations (*p* < 0.01) with most volatile flavor substances and major organic acids (especially SAFRANAL, Hexyl acetate, Ethyl lactate, Ethyl acetate, Lactic acid, and Acetic acid). *Candida* also displayed positive correlations (*p* < 0.01) with nearly all major volatile flavor substances and organic acids, with a significant correlation observed specifically for acetic acid. In the Lpscw group, *Saccharomyces*, *Candida*, *Aspergillus*, and *Microglena* were positively correlated with major volatile flavor substances, whereas *Pichia* did not show any significant positive correlations. *Candida*, *Aspergillus*, and *Microglena* demonstrated positive correlations with the majority of volatile flavor substances, with several genera among the top 10 fungi in relative abundance showing the ability to produce volatile flavor substances. This contrasts with the CK group, where most genera inhibited the production of volatile flavor substances.

## 3 DISCUSSION

Traditional fermented food production using the artisanal model often faces challenges related to safety and quality consistency, while industrial production may simplify the fermentation system, potentially affecting the flavor. Artificial inoculation of SynComs provides a potential solution to these challenges. Not all microorganisms in a fermentation system are essential, as some have redundant functions. By carefully selecting and combining microbial communities, the productivity and product quality consistency can be enhanced while retaining unique flavors. This approach requires advanced technologies to build, optimize, and validate SynComs for the industrial production of traditional fermented foods.

In our study, based on the identification method proposed by Wang Shilei et al. for the liquor brewing system, we correlated the changes in the content of volatile flavor substances‐including major esters, alcohols, non‐volatile flavor acids, and free amino acids‐with the microbial community data [28]. We selected species with strong positive correlations to various flavor substances. Then we intersected those involved in acid and ester production to identify the microbial genera capable of producing both. Finally, the results of microbial correlation network analysis were integrated to identify the strong node microorganisms in the microbial community of zha‐chili, and these were further intersected with the flavor‐producing microbial genera to determine the statistically significant “core microbial community.” The results from the CK group, which was subsequently inoculated with SynComs, demonstrated that the fungal genera in the “final core microbial community” consistently acted as flavor‐producing microbial genera in the CK group. This consistency across replicated experiments of zha‐chili fermentation over 2 years, even without inoculation or special treatments, which aligns with our previous study. These results suggest that our method for screening the “core microflora” of zha‐chili is reliable.

Through single‐strain inoculation experiments, we found that the trends and ranges of moisture content, pH, acidity, and reducing sugar content were consistent across all experimental groups. Similarly, the effect of organic acids produced by single‐strain fermentation was not effective enough. Regarding the impact on flavor substances, the LAB and Wess groups were superior to others in producing ethyl lactate and ethyl acetate, while the *Pichia*‐inoculated group exhibited a higher saffron aldehyde content. However, the five experimental groups inoculated with fermenting strains did not significantly outperform the uninoculated CK group. Although some single‐strain inoculated groups enhanced the flavor of zha‐chili to some extent, their overall impact on flavor production was not substantially better than that of the CK group. This finding underscores the necessity of conducting inoculated fermentation with SynComs.

SynComs inoculation led to an overall improvement in the quality of zha‐chili. Firstly, in terms of changes in physicochemical factors and organic acids, the within‐group differences in the Lpscw group were smaller than in the CK group, indicating more stable changes in physicochemical factors and organic acid production in the Lpscw group. Secondly, the peak organic acid content in the Lpscw group generally occurred on the 30th day, suggesting that inoculation with SynComs could shorten the fermentation time of zha‐chili. Regarding the content of volatile flavor substances, the levels of ethyl acetate and ethyl lactate in the Lpscw group increased by 8% compared to the CK group. Additionally, the levels of major organoleptic substances in the Lpscw group reached their maximum on the 30th day, whereas the CK group generally reached this maximum on the 45th day. This suggests that the synthetic microbial community can also shorten the production cycle of fermented flavor products and increase the levels of volatile organoleptic substances. Notably, regarding functional gene composition, inoculation with SynComs altered the abundance of the GH (glycoside hydrolases) family during the fermentation of zha‐chili. This may indicate that SynComs inoculation changed the types of carbohydrate‐associated catabolic enzymes within the microbial community, thereby affecting the community's ability to metabolize carbohydrates and altering the types of metabolites produced during flavor synthesis.

SynComs affected the eukaryotic microbial composition of zha‐chili fermentation communities. Combined with the Raup‐Crick phasor index analysis, the RC indices of both the Lpscw and CK groups were close to −1, suggesting that the succession of bacterial communities was predominantly driven by deterministic processes [36]. This suggests the presence of strong ecological filters within the zha‐chili fermentation ecosystem, imposing strict constraints on bacterial communities. In terms of fungal community succession, most of the RC indices of the CK group were close to −1, indicating that the fungal communities were more similar and that differences between them being smaller than expected by chance, largely due to deterministic influences. However, the fungal community succession in the Lpscw group differed, with some RC values near 0, indicating that the degree of similarity (or dissimilarity) was closer to the predictions of the neutral model [37, 38]. Additionally, samples from the Lpscw group on Day 45 differed from those on Day 0, implying that environmental ecological filters were less restrictive on the Lpscw group than the CK group.

SynComs may improve the overall microbial system by influencing the functions carried out by other microorganisms in the community. The results of the correlation analysis between eukaryotic microorganisms and the primary flavor compounds in the CK group were consistent with those from the SynComs screening phase. Specifically, the genera *Pichia, Saccharomyces*, and *Candida* showed strong positive correlations with the major flavor substances. Despite a 2‐year gap between the two sets of experiments, the results remained consistent, confirming the reliability of the SynComs we screened. Interestingly, *Pichia*, which dominated the Lpscw group, did not show a correlation with the primary flavor compounds strongly. In contrast, other fungi among the top 10 in relative abundance positively contributed to the main flavor profile. This suggests that the role of inoculated SynComs extends beyond flavor production by the inoculated fungi, enhancing the contribution of other microbes in the community to the overall flavor profile. Based on the observed changes in the successional pattern of the Lpscw fungal community and the significant alterations in the composition of the GH family, we infer that SynComs inoculation likely operates at the community level, enhancing the contribution of high‐abundance microorganisms to flavor compound production.

## 4 CONCLUSION

In this study, we screened and established the SynComs associated with the fermentation of zha‐chili, and verified the inoculation effect of the SynComs in subsequent single‐organism and mixed‐organism fermentation experiments. We investigated the changes in microbial community composition and functional gene composition during the fermentation process by macro‐genome sequencing and monitored the changes in volatile flavor compounds during the fermentation process by GC‐MC. The improvement of zha‐chili by SynComs reduced the fermentation time by about 15 days, and increased the yields of the main flavors (e.g., 8% for both ethyl lactate and ethyl acetate), which provides important guidance for the industrial‐scale production of zha‐chili. Meanwhile, SynComs altered microbial communities' composition and succession patterns in the zha‐chili fermentation system, especially affecting the fungal community so that *Pichia* became the dominant microorganism throughout the fermentation process. There were also changes in the functional gene composition, particularly in the abundance of the GH family. These findings highlight the important role of SynComs in improving traditional naturally fermented foods, which could provide an effective approach to improving the safety and flavor stability of fermented foods in the future. The study suggests that SynComs can improve the functionality of the entire microbial community by modulating the roles of other microbial species. This inference should be further validated through studies on additional microbial systems, which will be a key objective of our future research.

## 5 METHODS

### 5.1 Experimental design and sample collection

Zha‐chili is a traditional fermented food made from corn (or rice) and fresh chili peppers, produced in a controlled, semi‐open environment. Sufficient seed culture was prepared for fermentation experiments. The strains obtained by screening were activated and cultured for two rounds, and the Sanger sequencing method was used to confirm the purity of the strains. We simulated the production process of zha‐chili in the laboratory by crushing dried chilies to make chili paste, mixing them well with corn flour in a ratio of 4:6, and then loading them into anaerobic respiratory bags for anaerobic fermentation. The production process is shown in Figure S1A.

The strains used to construct SynComs were obtained from in situ screening of products from different stages of zha‐chili fermentation through the spread plate method. MRS medium (with color developer), YPD medium, LB medium, and Rose Bengal Medium were used (formulations in Table S2). After several rounds of screening, the native strains in zha‐chili were obtained. We screened many zha‐chili fermentation products in 3 rounds, but only some of the indigenous strains screened from zha‐chili were obtained.

We inoculated the logarithmic phase, and the number of bacteria in the logarithmic phase of each strain is shown in Table S3. Each 500g of fermentation sample was inoculated with 5 mL of concentrated suspension (inoculum concentration of 10^6^ cfu/g); Similarly, 5 mL of mixed suspension was inoculated per 500 g of sample in the mixed bacterial fermentation group, with the ratio of the five bacterial strains being 1:1:1:1:1 (inoculum concentration of 10^6^ cfu/g suspension Before inoculation, sufficient ddH_2_O was prepared in place of the medium combination to remove the effects of the medium, and the suspension was concentrated by centrifugation (4000 × *g*, 5 min).

Samples were collected from each batch at 0, 3, 10, 30, and 45 days of fermentation, three parallels were taken at each time point, and more than five replicates of each sample were kept for backup. Fermentation zha‐chili samples were randomly selected from the total fermentation samples and mixed well before sampling. Some samples were collected for physicochemical property analyses, and the rest were placed in an ultra‐low temperature refrigerator at −80°C for cryopreservation for subsequent experiments. The sample information is shown in Table S4.

### 5.2 Physicochemical factors analysis

The moisture, pH, acidity, and reducing sugar contents of zha‐chili during fermentation were detected based on our previous studies [21]. When measuring moisture content, 4 g of sample was dried at 105°C for 3 h to a constant weight and calculated as weight loss divided by the original weight. As for assessing pH and acidity, 5 g of sample was added into 50 mL of ddH_2_O with shaking at 150 rpm for 30 min, then the supernatant after 30 min of static was used for measuring pH value and acidity by pH meter and acid–base titration, respectively. The reducing sugar content of the sample was determined by 3, 5‐dinitro salicylic acid according to the standard reducing sugar solution.

### 5.3 Flavor substances analysis

As for organic acids and aroma compounds, 5 g of sample was suspended in 20 mL of ddH_2_O, ultrasonicated at 0°C for 30 min, then centrifuged at 8000 rpm for 10 min at 4°C, and the supernatant was filtered with 0.22‐μm filter for analysis. Acetic acid, lactic acid, and ethanol contents were separated in a Bio‐Rad HPX‐87H column (300 × 7.8 mm) at 40°C with a constant flow rate of 0.6 mL/min in isocratic elution (5 mM H_2_SO_4_) and then detected by refractive index detector (RID) at 35°C coupled in a high‐performance liquid chromatography (HPLC, Agilent 1200) system [39]. A head‐space solid‐phase microextraction and gas chromatography‐mass spectrometry (HS‐SPMEGC‐MS) technique was used to detect other aroma compounds based on Sun's study [39]. Namely, 8 mL of the above‐filtered supernatant was put into a 20‐mL autosampler vial with 3.0 g of NaCl to get a saturated NaCl solution (the internal standard substances of 2‐octanol and 2‐ethyl hexanol were added). Then, the solution was extracted by an automatic headspace sampling system (Multipurpose Sample MPS 2XL) at 50°C for 45 min. After that, the SPME fiber was inserted into the injection port at 250°C for 5 min, and the compounds in the sample were separated via Agilent HP‐5 column (30 m × 0.25 mm i.d.; 0.25 μm film thickness) and DB‐FFAP (Decoary Bonded Fluorinated‐Fused Silica Apolar) column (60 m × 0.25 mm i.d.; 0.25 μm film thickness) and analyzed via GC‐MS (Agilent 7890B GC system and 5977C mass selective detector). Subsequently, the compounds were identified by comparing their mass spectral profiles (match quality ≥ 80) in the National Institute of Standards and Technology database.

### 5.4 Metagenome DNA extraction and shotgun sequencing

Total microbial genomic DNA samples were extracted using the OMEGA Mag‐Bind Soil DNA Kit (M5635‐02) (Omega Bio‐Tek), following the manufacturer's instructions, and stored at −20°C before further assessment. The quantity and quality of extracted DNAs were measured using a Qubit™ 4 Fluorometer, with WiFi Q33238 (Qubit™ Assay Tubes: Q32856; Qubit™ 1X dsDNA HS Assay Kit: Q33231) (Invitrogen) and agarose gel electrophoresis, respectively. The extracted microbial DNA was processed to construct metagenome shotgun sequencing libraries with insert sizes of 400 bp by Illumina TruSeq Nano DNA LT Library Preparation Kit. Each library was sequenced by the Illumina NovaSeq platform (Illumina) with the PE150 strategy at Personal Biotechnology Co., Ltd.

### 5.5 Metagenomics analysis

Raw sequencing reads were processed to obtain quality‐filtered reads for further analysis. First, sequencing adapters were removed from sequencing reads using Cutadapt (v1.2.1). Secondly, low‐quality reads were trimmed using a sliding‐window algorithm in Fastp [40]. Once quality‐filtered reads were obtained, taxonomical classifications of metagenomics sequencing read from each sample were performed using Kraken2 [41] against a RefSeq‐derived database, which included genomes from archaea, bacteria, viruses, fungi, protozoans, metazoans, and viridiplantae. Reads assigned to metazoans or viridiplantae were removed for downstream analysis. Megahit (v1.1.2) [42] assembled each sample using the meta‐large presetted parameters. The generated contigs (longer than 300 bp) were then pooled together and clustered using mmseqs.2 (Steinegger and SöDing 2017) with “easy‐linclust” mode, setting the sequence identity threshold to 0.95 and covering residues of the shorter contig to 90%. The lowest common ancestor taxonomy of the nonredundant contigs was obtained by aligning them against the NCBI‐nt database by mmseqs.2 [43] with “taxonomy” mode, and contigs assigned to Viridiplantae or Metazoa were dropped in the following analysis. MetaGeneMark [44] was used to predict the genes in the contigs. CDS sequences of all samples were clustered by mmseqs.2 with “easy‐cluster” mode, setting the protein sequence identity threshold to 0.90 and covering residues of the shorter contig to 90%. To assess the abundance of these genes, the high‐quality reads from each sample were mapped onto the predicted gene sequences using salmon in the quasi‐mapping‐based mode with “‐‐meta ‐‐minScoreFraction = 0.55”, and the copy per kilobase per million mapped reads (CPM) was used to normalize abundance values in metagenomes. The nonredundant genes' functionality was obtained by annotating using mmseqs with the “search” mode against the KEGG EggNOG and CAZy databases' protein databases, respectively. EggNOG and GO were obtained using EggNOG‐mapper (v2) [45]. GO ontology was obtained using map2slim (www.metacpan.org). KO was obtained using KOBAS [46].

### 5.6 Statistical analysis

Data manipulation and visualization were performed through the R meta package tidyverse (2.0.0) [47]. *T*‐test, Kruskal–Wallis rank sum test, and Wilcoxon rank sum test were performed through functions “t.test,” “kruskal.test,” and “wilcox.test” in package stats (4.2.1). Before analysis, the samples were rarefied to uniform depth based on the lowest sample sequence to eliminate the influence of different sequencing depths. Alpha diversity indices (Shannon, pielou's eveness, observed species, and faith's pd) were calculated using functions diversity in vegan (2.7) [48]. Beta diversity metrics (Jaccard dissimilarity, Bray‐Curtis dissimilarity), as well as PCA, were conducted using the function “rda” in vegan (2.7) [49] followed by an ADNOIS test to measure the changes related to sampling sites [50]. Heatmaps were plotted using the complexheatmap (2.16.0) and pheatmap (1.012) packages [51]. The Raup–Crick dissimilarity index was calculated using a custom function provided by Chase et al. All visualizations are done in R, mainly based on ggplot2 (3.5.1).

## AUTHOR CONTRIBUTIONS

**Hongye Shen**: Conceptualization; methodology; writing—original draft; writing—review and editing; investigation; software; visualization; project administration, resources; data curation. **Chuanyu Du**: Conceptualization; methodology; investigation. **Shu Jiang**: Conceptualization; investigation; methodology. **Weiwei Dong**: Writing—original draft; writing—review and editing. **Jinshan Li**: Writing—original draft; writing—review and editing. **Yongmei Hu**: Writing—original draft; writing—review and editing. **Nan Peng**: Writing—original draft. **Shumiao Zhao**: Funding acquisition; conceptualization; investigation; methodology; writing—review and editing; writing—original draft; project administration.

## CONFLICT OF INTEREST STATEMENT

The authors declare no conflicts of interest.

## 1 ETHICS STATEMENT

The authors have nothing to report.

## Supporting information

The online version contains supplementary figures and tables available.

**Figure S1.** Overview of the workflow.
**Figure S2.** Results of LEfSe analyses between Lpscw and CK group communities.
**Figure S3.** Functional gene composition of the CK and Lpscw groups.
**Figure S4.** Results of LEfSe analysis between functional genes in Lpscw and CK groups.

**Table S1.** Genera included in each functional group.
**Table S2.** Formulation of the media used in the experiment.
**Table S3.** Number of viable bacteria per unit of bacterial solution.
**Table S4.** Sample collection time and sample naming information.

## Data Availability

The data that support the findings of this study are available from the corresponding author upon reasonable request. All amplicon sequencing raw data have been deposited in the NCBI Sequence Read Archive database under the BioProject ID PRJNA782862 (https://www.ncbi.nlm.nih.gov/bioproject/PRJNA782862/) and PRJNA784134 (https://www.ncbi.nlm.nih.gov/bioproject/PRJNA784134/). All the metagenomic sequencing raw data have been deposited in the NCBI Sequence Read Archive database under BioProject number PRJNA1131906 (https://www.ncbi.nlm.nih.gov/bioproject/PRJNA1131906/). The data and scripts used in this study can be found at https://github.com/hyShen-hzau/Synthetic-Microbial-Communities-in-iMateOmics.git. Supplementary materials (figures, tables, graphical abstract, slides, videos, Chinese translated version, and update materials) may be found in the online DOI or iMetaOmics http://www.imeta.science/imetaomics/.
