# Supplementary material for: Native synthetic microbial communities enhance zha‐chili by boosting the fermentation capacity of indigenous microorganisms
Source: IMetaOmics. 2025 Mar 16;2(2):e70009. doi: 10.1002/imo2.70009 (PMC12806400; doi:10.1002/imo2.70009)
Supplement: Supplementary file 1 — Figure S1. Overview of the workflow. Figure S2. Results of LEfSe analyses between Lpscw and CK group communities. Figure S3. Functional gene composition of the CK and Lpscw groups. Figure S4. Results of LEfSe analysis between functional genes in Lpscw and CK groups. [file IMO2-2-e70009-s002.docx]

**Supporting information to**

**Native Synthetic Microbial Communities Enhance zha-chili Fermented Foods by Boosting the Fermentation Capacity of Indigenous Microorganisms**

**Running title: Construction of synthetic microbial communities in zha-chili**

Hongye Shen^1^, Chuanyu Du^1^, Shu Jiang^1^, Weiwei Dong^2^, Jinshan Li^1^, Yongmei Hu^1^, Nan Peng^1^, Shumiao Zhao^1#^

^1^National Key Laboratory of Agricultural Microbiology and College of Life Science and Technology, Huazhong Agricultural University, Wuhan 430070, China

^2^College of Life Sciences, Hubei Normal University, Huangshi 435002, China

^#^Correspondence: [shumiaozhao@mail.hzau.edu.cn](mailto:shumiaozhao@mail.hzau.edu.cn) (Shumiao Zhao)


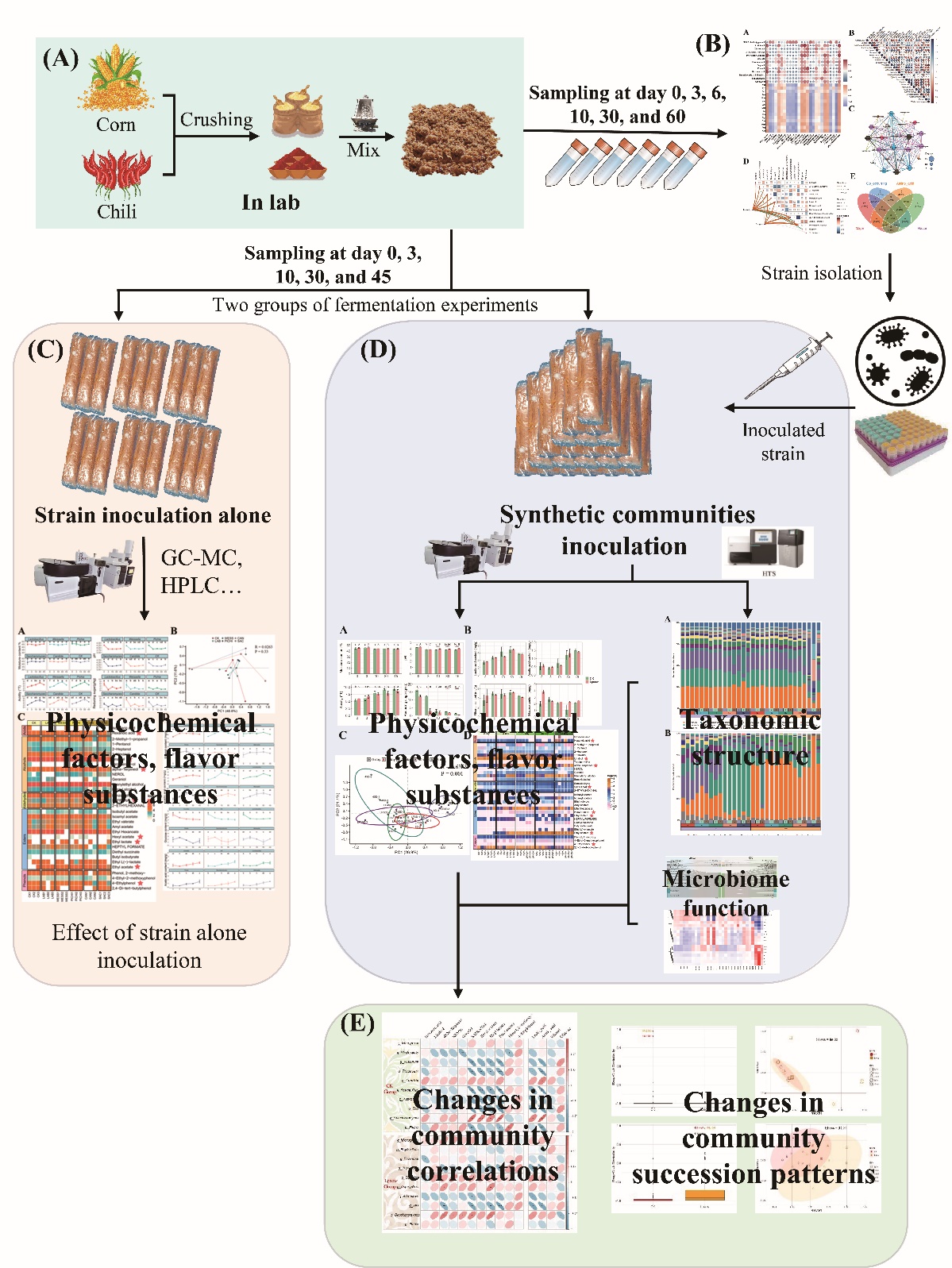


**Figure S1 Overview of the workflow.** (A) Process flow of zha-chili production reproduced in the laboratory. (B) Statistically significant establishment of synthetic microbial communities and isolation of strains in zha-chili. (C) Verification of fermentability of individual strains in the synthetic microbial community, (D) Verification of fermentability of synthetic microorganisms. (E) Impact of synthetic microbial communities on fermentation microcosm.

**
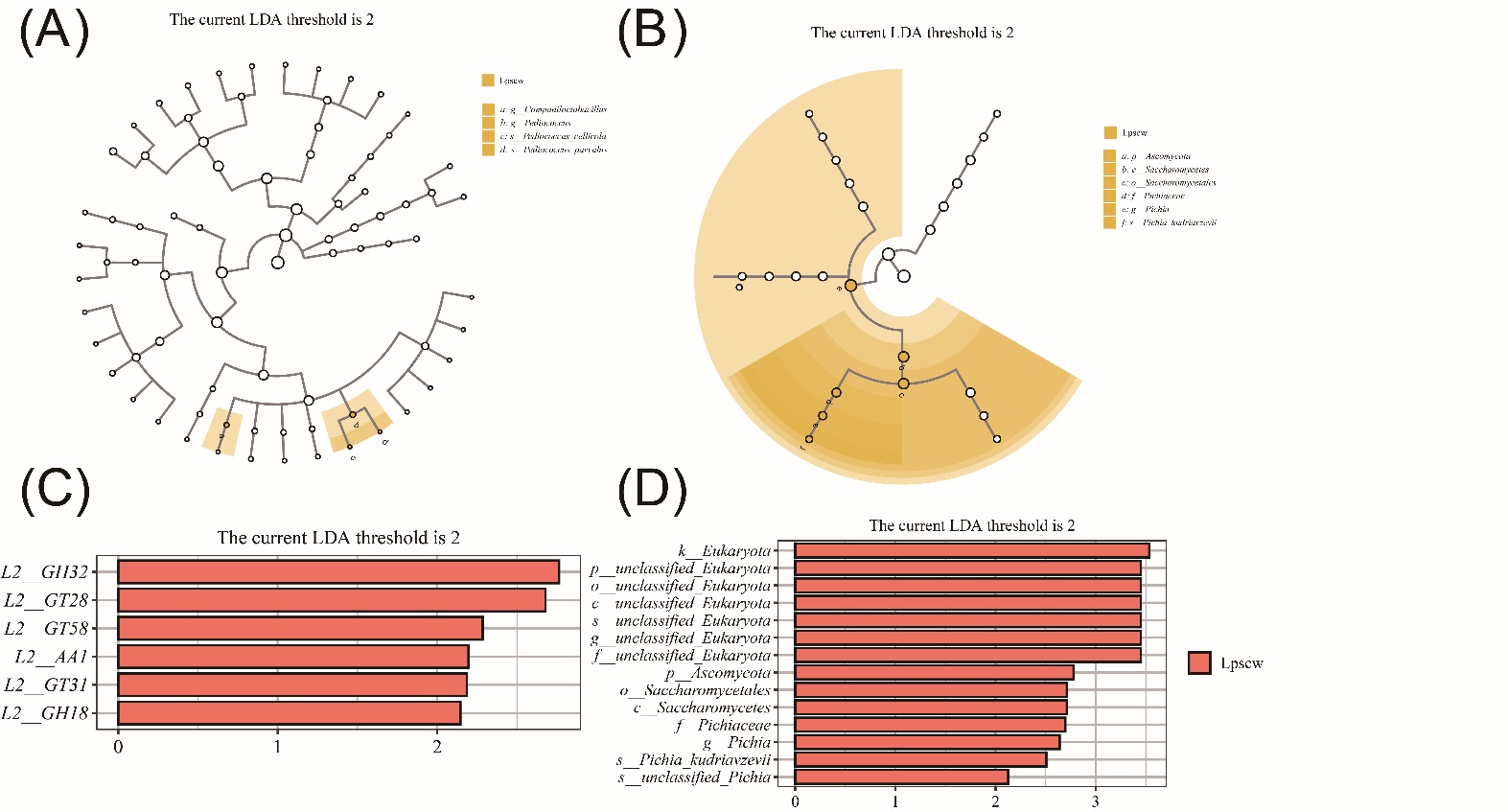
Figure S2 Results of LEfSe analyses between Lpscw and CK group communities.** (A), Evolutionary branching diagram for the bacterial community; (B), Evolutionary branching diagram for the fungal community; (C), Distribution histogram for the bacterial community; (D), Distribution histogram for the fungal community


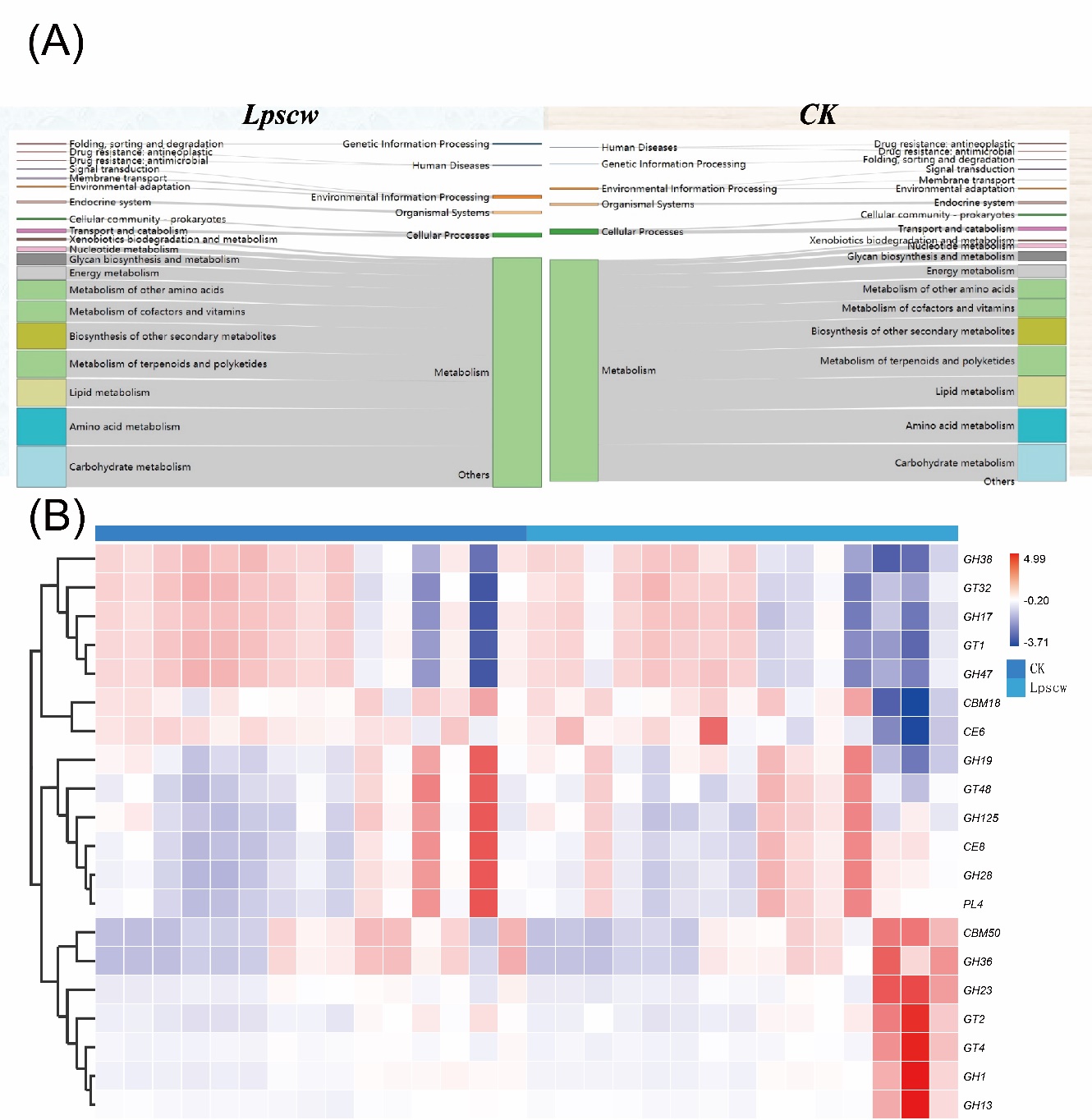


**Figure S3 Functional gene composition of the CK and Lpscw groups.** (A) Results of the first and second metabolic pathways based on the KEGG PATHWAY database annotations. (B) Annotation results of Family level of CAZy database during the fermentation process of zha-chili.


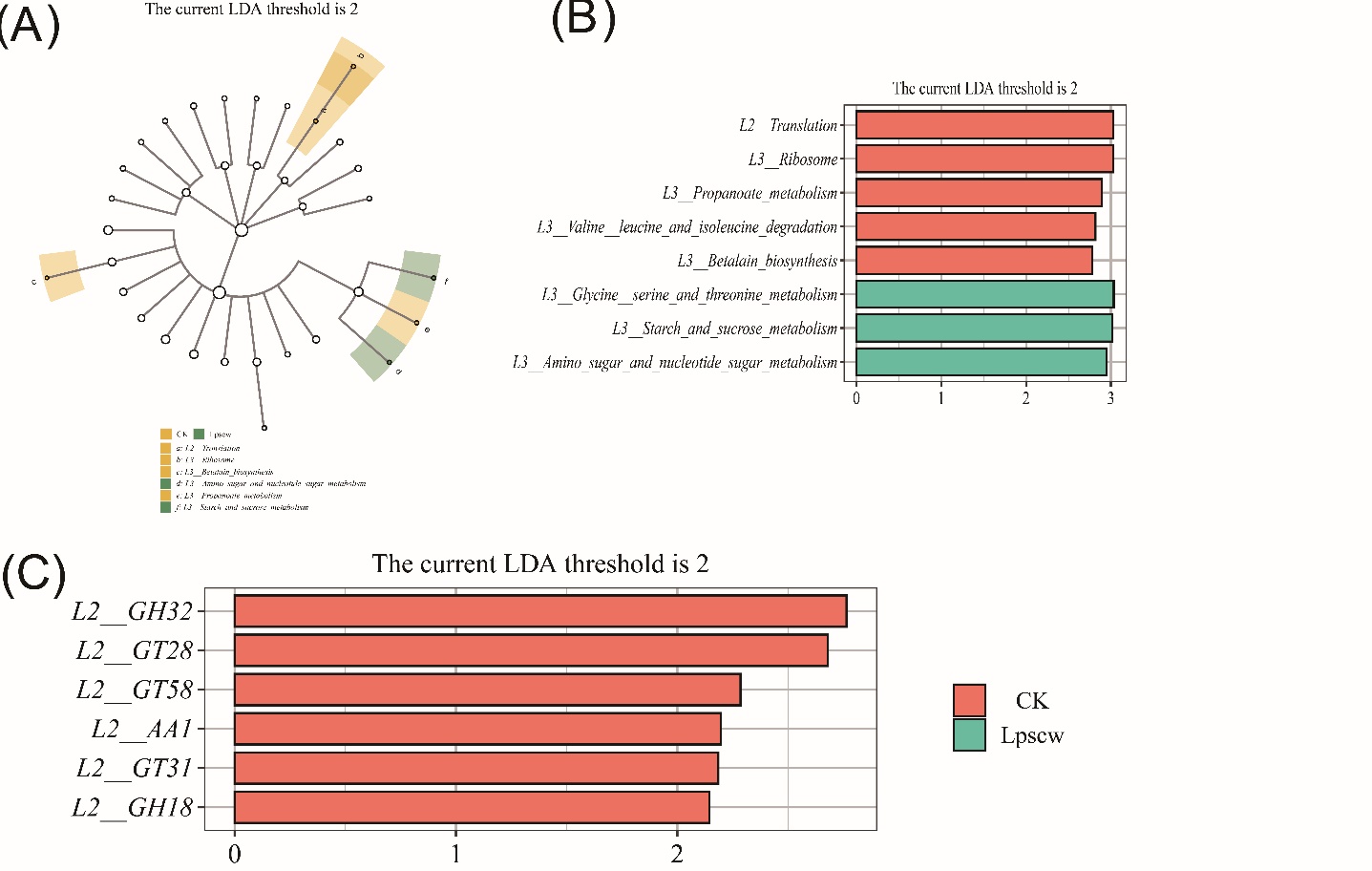


**Figure S4 Results of LEfSe analysis between functional genes in Lpscw and CK groups.** (A), Evolutionary branching diagram of KEGG annotation results; (B), Histogram of distribution of KEGG annotation results; C, Histogram of distribution of CAZy annotation results.
